# ESUR/ESUI consensus statements on multi-parametric MRI for the detection of clinically significant prostate cancer: quality requirements for image acquisition, interpretation and radiologists’ training

**DOI:** 10.1007/s00330-020-06929-z

**Published:** 2020-05-19

**Authors:** Maarten de Rooij, Bas Israël, Marcia Tummers, Hashim U. Ahmed, Tristan Barrett, Francesco Giganti, Bernd Hamm, Vibeke Løgager, Anwar Padhani, Valeria Panebianco, Philippe Puech, Jonathan Richenberg, Olivier Rouvière, Georg Salomon, Ivo Schoots, Jeroen Veltman, Geert Villeirs, Jochen Walz, Jelle O. Barentsz

**Affiliations:** 1 Department of Radiology & Nuclear Medicine and Anatomy, Radboud University Medical Center, Radboud Institute for Health Sciences, Nijmegen, The Netherlands; 2 Department of Urology and Department of Radiology & Nuclear Medicine, Radboud University Medical Center, Radboud Institute for Health Sciences, Nijmegen, The Netherlands; 3 Department for Health Evidence, Radboud University Medical Center, Radboud Institute for Health Sciences, Nijmegen, The Netherlands; 4 Imperial Urology, Charing Cross Hospital, Imperial College Healthcare NHS Trust, London, UK; 5 Division of Surgery, Department of Surgery and Cancer, Faculty of Medicine, Imperial College London, London, UK; 6 Department of Radiology, CamPARI Prostate Cancer Group, Addenbrooke’s Hospital and University of Cambridge, Cambridge, UK; 7 Department of Radiology, University College London Hospital NHS Foundation Trust, London, UK; 8 Division of Surgery & Interventional Science, University College London, London, UK; 9 Department of Radiology, Charité, Berlin, Germany; 10 Department of Radiology, Herlev Gentofte University Hospital, Herlev, Denmark; 11 Paul Strickland Scanner Centre, Mount Vernon Cancer Centre, Northwood, UK; 12 Department of Radiological Sciences, Oncology and Pathology, Sapienza University of Rome, Rome, Italy; 13 Department of Radiology, University of Lille, Lille, France; 14 Department of Imaging, Brighton and Sussex University Hospital NHS Trust, Brighton, UK; 15 Department of Urinary and Vascular Radiology, Hôpital Édouard-Herriot, Lyon, France; 16 Faculté de Médecine Lyon Est, Université Lyon 1, Lyon, France; 17 Martini Clinic, University Medical Center Hamburg-Eppendorf, Hamburg, Germany; 18 Department of Radiology & Nuclear Medicine, Erasmus MC University Medical Center, Rotterdam, The Netherlands; 19 Department of Radiology, Netherlands Cancer Institute, Amsterdam, The Netherlands; 20 Department of Radiology, Ziekenhuisgroep Twente, Almelo, The Netherlands; 21 Department of Radiology, Ghent University Hospital, Ghent, Belgium; 22 Department of Urology, Institute Paoli-Calmettes Cancer Center, Marseille, France

**Keywords:** Consensus, Diagnosis, Magnetic resonance imaging, Multi-parametric magnetic resonance imaging, Prostatic neoplasms

## Abstract

**Objectives:**

This study aims to define consensus-based criteria for acquiring and reporting prostate MRI and establishing prerequisites for image quality.

**Methods:**

A total of 44 leading urologists and urogenital radiologists who are experts in prostate cancer imaging from the European Society of Urogenital Radiology (ESUR) and EAU Section of Urologic Imaging (ESUI) participated in a Delphi consensus process. Panellists completed two rounds of questionnaires with 55 items under three headings: image quality assessment, interpretation and reporting, and radiologists’ experience plus training centres. Of 55 questions, 31 were rated for agreement on a 9-point scale, and 24 were multiple-choice or open. For agreement items, there was consensus agreement with an agreement ≥ 70% (score 7–9) and disagreement of ≤ 15% of the panellists. For the other questions, a consensus was considered with ≥ 50% of votes.

**Results:**

Twenty-four out of 31 of agreement items and 11/16 of other questions reached consensus. Agreement statements were (1) reporting of image quality should be performed and implemented into clinical practice; (2) for interpretation performance, radiologists should use self-performance tests with histopathology feedback, compare their interpretation with expert-reading and use external performance assessments; and (3) radiologists must attend theoretical and hands-on courses before interpreting prostate MRI. Limitations are that the results are expert opinions and not based on systematic reviews or meta-analyses. There was no consensus on outcomes statements of prostate MRI assessment as quality marker.

**Conclusions:**

An ESUR and ESUI expert panel showed high agreement (74%) on issues improving prostate MRI quality. Checking and reporting of image quality are mandatory. Prostate radiologists should attend theoretical and hands-on courses, followed by supervised education, and must perform regular performance assessments.

**Key Points:**

*• Multi-parametric MRI in the diagnostic pathway of prostate cancer has a well-established upfront role in the recently updated European Association of Urology guideline and American Urological Association recommendations*.

*• Suboptimal image acquisition and reporting at an individual level will result in clinicians losing confidence in the technique and returning to the (non-MRI) systematic biopsy pathway. Therefore, it is crucial to establish quality criteria for the acquisition and reporting of mpMRI*.

*• To ensure high-quality prostate MRI, experts consider checking and reporting of image quality mandatory. Prostate radiologists must attend theoretical and hands-on courses, followed by supervised education, and must perform regular self- and external performance assessments*.

## Introduction

Multi-parametric MRI (mpMRI) in the diagnostic pathway of prostate cancer (PCa) has a well-established upfront role in the recently updated European Association of Urology (EAU) guideline and American Urological Association recommendations [1, 2]. For biopsy-naïve men with suspicion of PCa, based on an elevated serum prostate–specific antigen level or abnormal digital rectal examination, it is now recommended to undergo a mpMRI before biopsy. Incorporation of mpMRI in the diagnostic pathway of men with clinical suspicion of PCa has several advantages compared to a systematic transrectal ultrasonography–guided biopsy (TRUSGB) approach. MRI can rule out clinically significant (cs)PCa and, therefore, will result in fewer unnecessary prostate biopsies [3–5]. Also, mpMRI reduces overdiagnosis and overtreatment of low-grade cancer [5–9]. Finally, mpMRI allows targeted biopsies of those lesions assessed as suspicious, enabling better risk stratification [10].

If one wants to take advantage of the ‘MRI pathway’, annually 1,000,000 men in Europe need to have a pre-biopsy MRI [11]. Performing such a high number of mpMRIs with high-quality acquisition and high-quality reporting is a significant challenge for the uroradiological community. Fortunately, the recently updated Prostate Imaging-Reporting and Data System (PI-RADS) version 2.1 defines global standardization of reporting and recommends uniform acquisition [12]. However, there is a lack of consensus on how to assure and uphold mpMRI acquisition and reporting quality. There is also a need to define requirements for learning and accumulation of reporting experience for mpMRI.

Suboptimal image acquisition and reporting at an individual level will result in clinicians losing confidence in the technique and returning to the (non-MRI) TRUS biopsy pathway. Therefore, it is crucial to establish quality criteria for both acquisition and reporting of mpMRI. Thus, this study aims to define consensus-based criteria for acquiring and reporting mpMRI scans and determining the prerequisites for mpMRI quality.

## Materials and methods

A Delphi consensus process was undertaken to formulate recommendations regarding three different areas in the diagnostic MRI pathway of PCa: (1) image quality assessment of mpMRI; (2) interpretation and reporting of mpMRI; and (3) reader experience and training requirements. The Delphi method is a technique of structured and systematic information gathering from experts on a specific topic using a series of questionnaires [13]. In this study, the diagnostic role of mpMRI in biopsy-naïve men with a suspicion of PCa was considered.

**Fig. 1:**
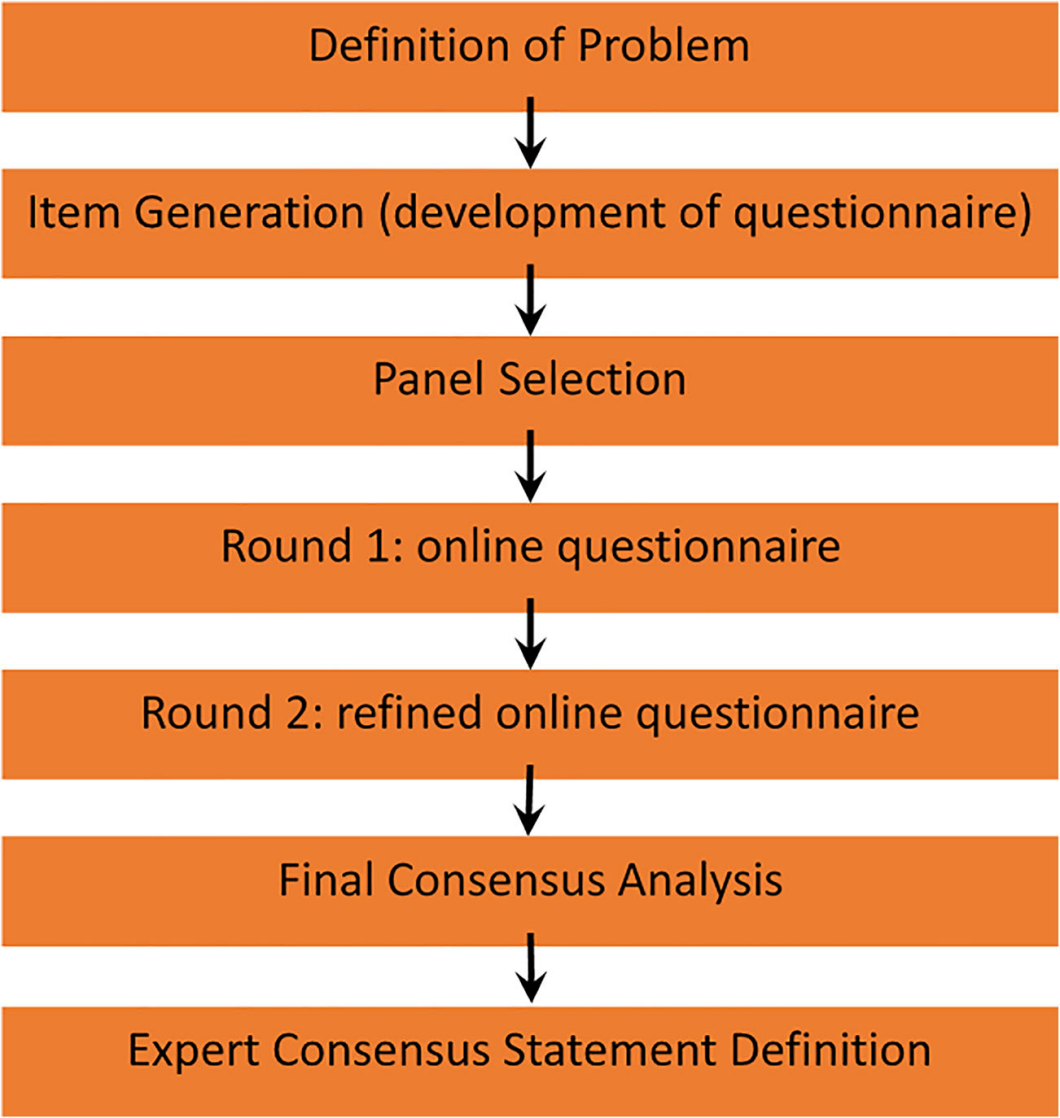
Details of the stages of the Delphi process (flow chart)

The Delphi process was carried out in four phases (Fig. 1). (1) Panellists from the European Society of Urogenital Radiology (ESUR) and EAU Section of Urologic Imaging (ESUI) were selected based on expertise and publication record in the PCa diagnosis, and on their involvement in guideline development. (2) A questionnaire was created with items that were identified by a subcommittee of the ESUR, based on the statements from a recent UK consensus paper on implementation of mpMRI for PCa detection [14]. (3) Panel-based consensus findings were determined using an online Delphi process. For this purpose, an internet survey was generated and sent by email to the members of the group (created in Google Forms). In the second round, a reminder to complete the questionnaires was sent by email. The panellists anonymously completed two rounds of a questionnaire consisting of 39 items (including 55 subquestions). Based on the knowledge of the entire group’s responses in the first round, second round voting was performed. Outcomes of the multiple-choice and open questions were graphically displayed, so the results could be reflected before selecting a response in the second round. For inclusion in the final recommendations, each survey item required to have reached group consensus by the end of the two survey rounds. (4) The items of the questionnaires were analysed, and consensus statements were formulated based on the outcomes. In total, 31 of 55 items were rated for agreement on a 9-point Likert scale.

An item scored as ‘agree’ (score 7–9) by ≥ 70% of participants and disagree (score 1–3) by ≤ 15% constituted ‘consensus agreement’ for an item. An item scored as ‘disagree’ (score 1–3) by ≥ 70% of participants and agree (score 7–9) by ≤ 15% was considered as ‘consensus disagreement’. The other items (24 of 55) were multiple-choice or open questions and were presented graphically. For the multiple-choice or open questions to reach consensus, a panel majority scoring of ≥ 50% was required.

## Results

The response rate for both rounds was 58% (44 of 76). The final panel comprised 44 urologists and urogenital radiologists who are experts in prostate cancer imaging. After the first round, eight subquestions were deleted based on comments from the panellists in the free-text fields because they considered these items either a duplication or not relevant (questions: 8b, 9b, 10b, 16c, 16d, 19b, 26b, 32b; Tables 1, 2 and 3). All deleted subquestions were questions without consensus in the first round.

**Table 1:**
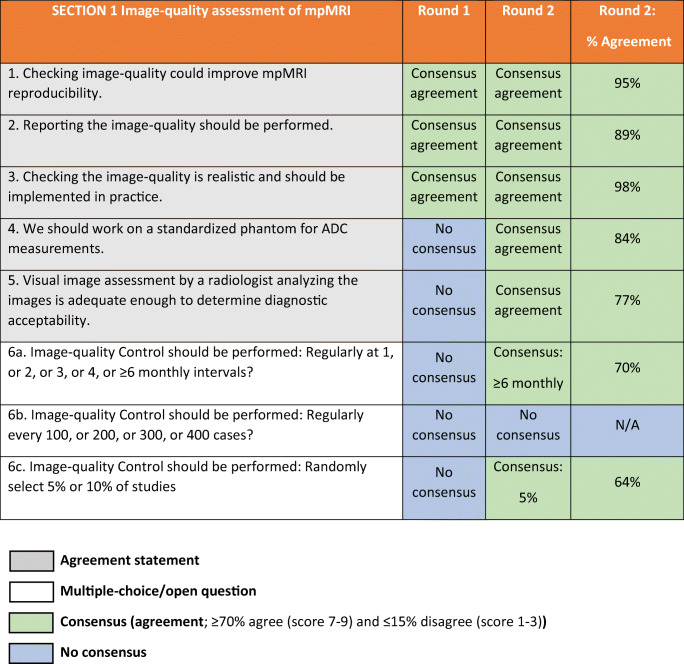
ESUR/ESUI consensus outcomes for section 1: Image quality assessment of mpMRI. *ADC* apparent diffusion coefficient, *ESUI* EAU Section of Urologic Imaging, *ESUR* European Society of Urogenital Radiology, *mpMRI* multi-parametric MRI, *N/A* not applicable

**Table 2:**
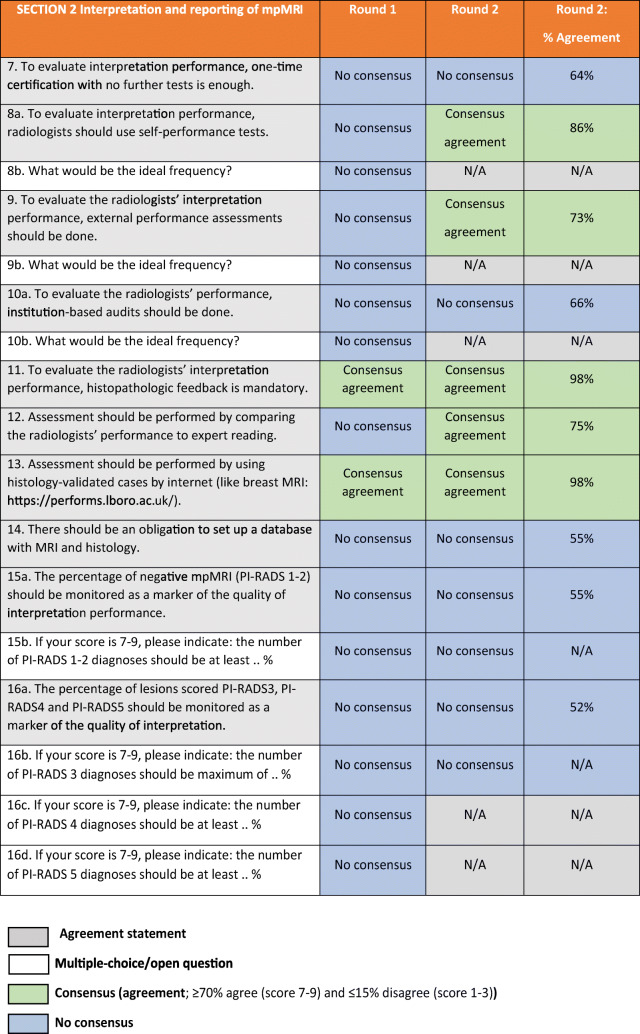
ESUR/ESUI consensus outcomes for section 2: Interpretation and reporting of mpMRI. *ADC* apparent diffusion coefficient, *ESUI* EAU Section of Urologic Imaging, *ESUR* European Society of Urogenital Radiology, *mpMRI* multi-parametric MRI, *N/A* not applicable

**Table 3:**
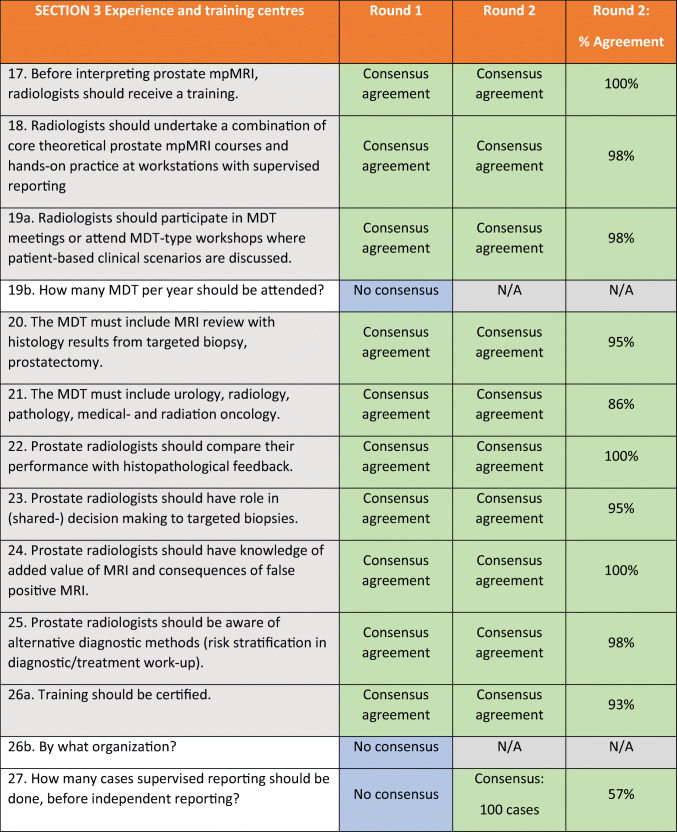

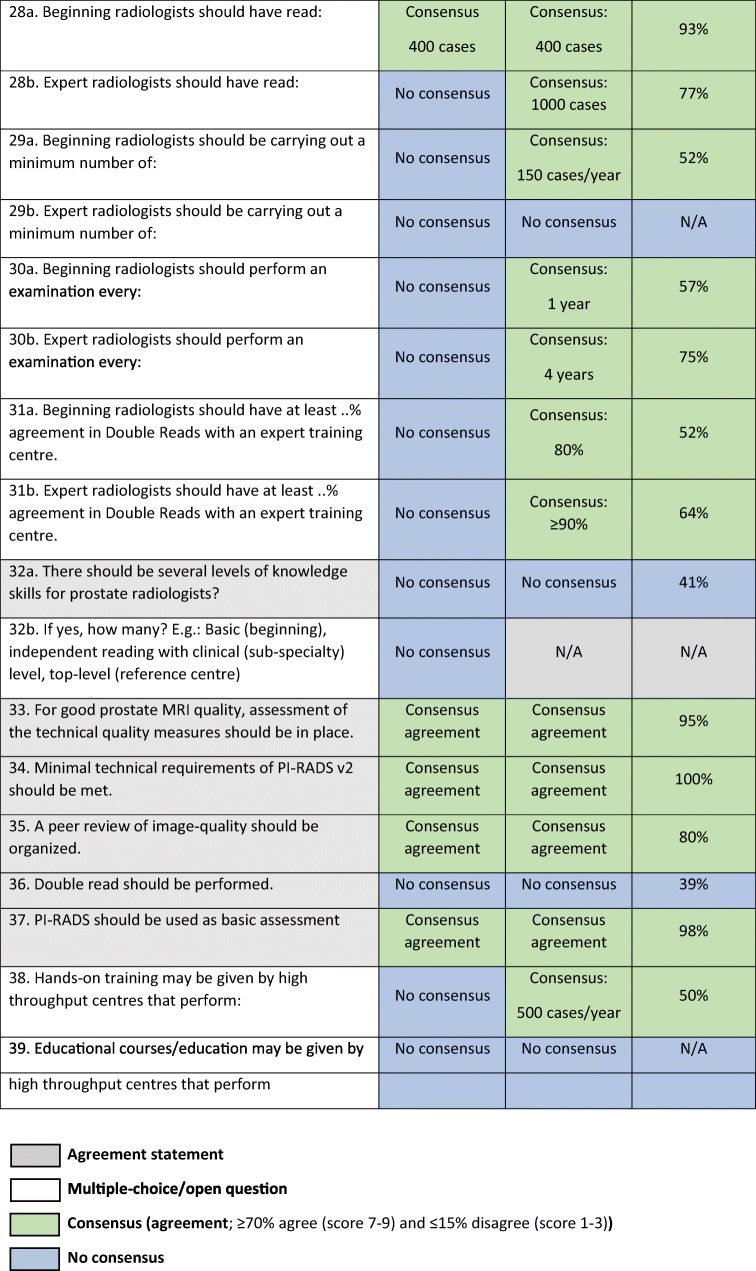
ESUR/ESUI consensus outcomes for section 3: Experience and training centres. *ESUI* EAU Section of Urologic Imaging, *ESUR* European Society of Urogenital Radiology, *MDT* multidisciplinary team, *mpMRI* multi-parametric MRI, *N/A* not applicable

After the first round of the Delphi process, consensus agreement was obtained in 19 of 31 (61%) questions that could be rated on a 1–9 scale. Consensus was obtained in 1 of 24 (4.2%) of multiple-choice/open questions. After the second round, this improved to 24 of 31 (77%) and in 11 of 16 (69%), respectively. None of the statements received consensual disagreement. Agreement statements combined with the outcomes of the multiple-choice/open questions were used to provide input for the recommendations regarding image quality and learning of prostate mpMRI and expertise of (training) centres (Tables 4 and 5).

**Table 4:** Consensus-based criteria ‘basic’ versus ‘expert’ radiologists. *N/A* not applicable

Basic	Criterion	Expert
100	Minimum number of supervised cases before independent reporting	N/A
400	Minimum number of cases read	1000
150	Minimum number of cases/year	200*
1	Examination interval (year(s))	4
80	Agreement in double reads with expert centre (%)	≥ 90

*No panel majority (most frequent answer 200 cases/year [41%; 18 of 44 panellists]; second most frequent answer was ≥ 500 cases/year [32%; 14 of 44 panellists])

**Table 5:** Consensus-based recommendations on image quality assessment (section 1), evaluation of interpretation performance (section 2) and reader experience with prostate MRI (section 3). *ADC* apparent diffusion coefficient, *MDT* multidisciplinary team, *mpMRI* multi-parametric MRI

Image quality	Interpretation performance	Reader experience
Checking and reporting the image quality should be performed.	To evaluate interpretation performance, radiologists should use self-performance tests.	Before interpreting prostate mpMRI, radiologists should receive training.
		Radiologists should undertake a combination of core theoretical prostate mpMRI courses and hands-on practice at workstations with supervised reporting.
		Training should be certified.
Visual image assessment by radiologists is adequate enough to determine diagnostic acceptability.	Assessment of radiologist performance should be performed using histopathologic feedback and by comparing to expert reading.	For good prostate MRI quality, assessment of the technical quality measures should be in place.
		A peer review of image quality should be organized.
		Minimal technical requirements of PI-RADS v2 should be met.
Image quality control should be performed ≥ 6 monthly or in 5% of studies.	To evaluate the radiologists’ interpretation performance, external performance assessments should be done.	PI-RADS should be used as the basis of assessments.
		Prostate radiologists should be aware of alternative diagnostic methods.
		Radiologists should participate in MDT meetings or attend MDT-type workshops.
		The MDT must include MRI review with histology results.
The radiologic community should work on a standardized phantom for apparent diffusion coefficient (ADC) measurements.		The MDT must include urology, radiology, pathology and medical and radiation oncology.
		Prostate radiologists should have knowledge on the added value of MRI and consequences of false results.
		Prostate radiologists should have roles in shared decision-making with respect to biopsy strategies.

### Section 1: Image quality assessment

The panellists consensually agreed on all five agreement statements in this section (Table 1). Consensus was reached on 2 out of 3 multiple-choice questions. Assessment of the technical image quality measures should be checked (question (Q)1) and reported (Q2), which can be qualitatively done by visual assessments by radiologists (Q5). Checking image quality is realistic and should be implemented into clinical practice (Q3). A majority of the panellists voted for external and objective image quality assessment regularly at 6 months or longer intervals (70%; 31 of 44 panellists). There was no consensus on whether image quality assessment was to be performed after a set number of cases, and panellists chose an interval of 300 or ≥ 400 cases in 25% (11 of 44 panellists) and 41% (18 of 44), respectively. Image quality checks could also be performed on a randomly selected sample of cases, wherein a majority (64%; 28 of 44 panellists) agreed that a selection of 5% of exams is most appropriate, but commented this could be dependent on the number of cases per centre.

Furthermore, the use of a standardized phantom for apparent diffusion coefficient value measurements is advocated (Q4), to enable quantifiable apparent diffusion coefficient (ADC) values that could be used as a threshold for the detection of csPCa in the peripheral zone.

### Section 2: Interpretation and reporting of mpMRI

The panellists have reached a consensus on 5/10 statements in this section (Table 2). There was no consensus on the multiple-choice questions in this section.

There was agreement on the use of self-performance tests to evaluate a radiologists’ performance (Q8a). Panellists did not agree upon the ideal frequency for this evaluation (Q8b; only answered in round 1). Consensus was reached on making use of histopathologic feedback, which is mandatory to evaluate the radiologists’ interpretation performance (Q11). Also, consensus was reached; comparing the radiologists’ performance to expert-reading (Q12), the use of external performance assessments (Q9a), and the use of internet-based histologically validated cases (Q13) should be part of the quality assessment, to improve individual radiologists’ skill at interpretation.

There was no consensus on using institution-based audits as part of the quality assessment on acquisition and reporting (Q10a). Also, there was no consensus on the use of a percentage of non-suspicious mpMRI (PI-RADS 1 or 2) as a marker for the quality of reporting (Q15a); the use of a percentage of PI-RADS 3, 4 or 5 as a marker for the quality of interpretation (Q16a); and on the questions about monitoring the percentages of PI-RADS 1–2 (non-suspicious), 3 (equivocal) or 4–5 (suspicious) lesions as markers for the quality of scan interpretations. Multiple panellists commented that the percentages in Q15 and Q16 are highly dependent on the prevalence of csPCa in the population at risk. There was no agreement on impelling a database with MRI and correlative histology mandatory (Q14).

### Section 3: Experience and training centres

This section comprised questions regarding general requirements for radiologists who interpret prostate mpMRI and statements on knowledge levels and experience (Table 3). Consensus was reached on 14 out of 16 agreement statements (88%) and 9 out of 11 multiple-choice/open questions (82%).

### General requirements

Before independently reading prostate mpMRI, radiologists should undertake a combination of core theoretical prostate mpMRI courses with lectures on the existing knowledge about prostate cancer (imaging) and hands-on practice at workstations where experts supervise reporting (Q17). The panellists agreed upon certification of training (Q26a). However, there was no consensus on what body (national or European) should be the certifying organization (Q26b). For good prostate mpMRI quality, assessment of the technical quality measures should be in place (Q33), and minimal technical requirements according to PI-RADS v2 should be met (Q34). Panellists agreed that peer reviews of image quality should be organized (Q35). PI-RADS should be used as a basic assessment tool (Q37). There was no consensus about impelling double-reading (Q36).

A prerequisite for radiologists who interpret and report prostate mpMRI should be that they participate in the multidisciplinary team (MDT) meetings or attend MDT-type workshops where patient-based clinical scenarios are discussed (Q19a). There was no agreement on the number of MDT meetings that should be attended per year. An MDT must include mpMRI review with histology results from the biopsy and, if performed, radical prostatectomy specimens (Q20) and presence of representatives from the urology, radiology, pathology and medical and radiation oncology departments (Q21). Prostate radiologists should have roles in the MDT in shared decision-making on (how to perform) targeted biopsy (Q23). Within this MDT, they should be aware of alternative diagnostic methods (risk stratification algorithms in diagnostic and treatment work-up) (Q25). Prostate radiologists should know the added value of mpMRI and the consequences of false positive or false negative mpMRI (Q24).

### Knowledge levels

There was no consensus about introducing several knowledge levels for prostate radiologists (Q32a), for instance, general (basic), good clinical (subspeciality) and top-level (reference centre). The panellists answered multiple-choice questions about the experience requirements for ‘basic’ versus ‘expert’ prostate radiologists and reached consensus on 8 out of 9 (sub)questions (see Table 4).

Novice prostate radiologists should begin with supervised reporting. A majority of the panellists favoured supervised reporting for at least 100 cases before independent reporting (57%; 25 of 44 panellists). In total, novice prostate radiologists should have read 400 cases to qualify as a ‘basic prostate radiologist’ (93%; 41 of 44 panellists). They should be carrying out a minimum of 150 cases/year (52%; 23 of 44 panellists) and perform an examination every year (57%; 25 of 44 panellists). In double-reads, basic prostate radiologists should have at least 80% agreement with an expert training centre read (52%; 23 of 44 panellists) on the assessment of PI-RADS 1–2 versus 3–5 lesions.

Expert prostate radiologists should have read at least 1000 cases (77%; 34 of 44 panellists). There was no consensus on how many exams an expert radiologist should read annually. Eighteen of 44 (41%) panellists favoured 200 cases/year, while 14 out of 44 (32%) panellists thought that expert radiologists should be carrying out a minimum of 500 cases/year. Expert radiologists should perform an examination every 4 years (75%; 33 of 44 panellists). They should have at least 90% agreement with an expert training centre read (64%; 28 of 44 panellists).

Fifty percent of the panellists (22 of 44 panellists) voted for at least 500 cases a year to give hands-on training. There was no consensus on the required number of cases per year a high-throughput centre should perform before being able to organize educational courses.

## Discussion

There is a lack of evidence on how to assess prostate mpMR image quality and on the requirements for those reading the examinations, including learning and experience prerequisites for independent reporting. This Delphi consensus documented by expert radiologists and expert urologists from the ESUR and the ESUI provides a set of recommendations to address these issues. They are offered as a starting point to improve the acquisition and reporting quality of mpMR images.

Three headings summarize the outcomes: (1) image quality assessment, (2) interpretation and reporting and (3) experience and training centres.

### Image quality assessment

There is a considerable variation in prostate MR image quality and compliance with recommendations on acquisition parameters. In a recent UK quality audit, 40% of patients did not have a prostate MRI that was adequate for interpretation, with a 38–86% compliance variation with recognized acquisition standards [12, 15–17]. The panellists agreed that reporting of image quality must be performed and implemented into clinical practice. Checking image quality was expected to improve mpMRI reproducibility. Before translating these recommendations into clinical practice, efforts are needed to develop qualitative and preferably also quantitative criteria to assess image quality.

### Interpretation and reporting

The panellists reached consensus on using self-performance tests, with histopathologic feedback, preferably compared to expert reading as well as to external performance assessments to determine individual radiologists’ reporting accuracy. A lower level of PI-RADS 3 cases (indeterminate probability of csPCa) is seen in expert centres compared to non-expert centres in biopsy-naïve men [7, 18]. However, the panel did not reach consensus on the use of cut-off levels for the various PI-RADS categories (1–2, 3 and 4–5). A minority of panellists favoured the use of a percentage as an indicator for the interpretation quality; most of them suggested a minimal PI-RADS 1–2 percentage of 20%, a maximum PI-RADS 3 percentage of 20–30% and a minimum percentage of PI-RADS 4 and PI-RADS 5 of 20–30% each. The high dependence of the PI-RADS distribution on the prevalence of csPCa is the reason for this lack of consensus. Nonetheless, in specifically defined populations, e.g. European biopsy-naïve patients (average csPCa prevalence of 25–40%), the percentage of PI-RADS 3 potentially is an indication of the ‘certainty’ of diagnosis and thus of image quality and reading. Recent studies show that differences of PI-RADS 3 rates (6–28%) are also attributable to magnetic field strength (1.5 versus 3 T, thus image quality), to strict adherence to the use of PI-RADS-assessment and of expert double-reading [7–9, 19, 20].

### Experience and training centres

There are scarce data that show a learning curve effect for mpMRI, the effect of a dedicated reader education program on PCa detection and diagnostic confidence and the effect of an online interactive case-based interpretation program [21–24]. Moreover, experienced urogenital radiologists show higher inter-reader agreement and better area under the receiver operating curve (AUC) characteristics as to radiologists with lower levels of experience [25–29]. In a relatively small sample size study, the AUC seems to remain stable after reading 300 cases but is significantly lower in readers who have read only 100 cases [27]. Nevertheless, thresholds for the number of prostate mpMRIs required before independent reporting and before reaching an expert level and the corresponding number of cases per year are not yet well established. Several previous studies suggested a dedicated training course followed by ≥ 100 expert-supervised mpMRI examinations [14, 22, 30]. For smaller centres or radiology groups that want to start a prostate MRI program, there are several existing (international) hands-on courses or possibilities to arrange (online) supervised readings by expert centres to facilitate this. The expert panel agreed that before interpreting mpMRI in addition to the recommendations in sections 1 and 2, a course should be attended, including theoretical and hands-on practice. Also, the expert panel listed a set of criteria for ‘basic’ and ‘expert’ prostate radiologists (Table 4). Radiologists should have read 100 supervised cases before independent reporting, have read a minimum of 300 cases before being classified as a ‘basic’ prostate radiologist and continue to read a minimum of 150 cases a year. For being classified as an ‘expert’ prostate radiologist, a minimum number of 1000 cases should be read. Also, there should be an examination every year for a novice prostate radiologist, and every 4 years for an expert. The panel did not reach a consensus on the number of cases a year an expert prostate radiologist should read (200/≥ 500 cases).

Some limitations need to be recognized. One of the limitations of a Delphi consensus process is that the results reflect the opinions of a selection of experts and are not based on a systematic literature review or meta-analyses. The methodology captures what experts think, and not what the evidence indicates in data-poor areas of practice. Also, definitions for consensus are arbitrary, and other definitions could result in different recommendations. The opinions of the expert panel can represent the intuition of experienced, knowledgeable practitioners who anticipate what the evidence would or will show, but they also can be wrong. Conflicts of interest can also influence expert opinion. However, as there are quite many participants (44), the influence of these biases is likely to be minimal. This modified Delphi process used a rigorous methodology in which questions were carefully designed. The consensus process and its results should be used for structuring the discussions of important topics regarding prostate MR image quality that currently lack evidence in the literature.

Because the questions addressed by the consensus are highly relevant for daily clinical practice, we are careful to emphasise that simply because experts agree does not mean they are right. Nevertheless, this consensus contributes to our knowledge. It captures what experts in the field think today regarding the need to implement reliable, high-quality prostate mpMRI as a diagnostic examination in the diagnostic pathway of biopsy-naïve men at risk of csPCa. This consensus-based statement should be used as a starting point, from where specific (reporting) templates will be developed, and future studies should be performed to validate the criteria and recommendations.

## Conclusion

This ESUR/ESUI consensus statement summarises in a structured way the opinions of recognized experts in diagnostic prostate mpMRI issues that are not adequately addressed by the existing literature. We focussed on recommendations on image quality assessment criteria and prerequisites for acquisition and reporting of mpMRI. Checking and reporting of prostate MR image quality are mandatory. Initially, prostate radiologists should have attended theoretical and hands-on courses, followed by supervised education, and must perform regular self- and external performance assessments, by comparing their diagnoses with histopathology outcomes.

## Abbreviations

csPCa: Clinically significant prostate cancer
EAU: European Association of Urology
ESUI: EAU Section of Urologic Imaging
ESUR: European Society of Urogenital Radiology
MDT: Multidisciplinary team
mpMRI: Multi-parametric MRI
PCa: Prostate cancer
Q: Question
TRUSGB: Transrectal ultrasound–guided biopsy
